# Collagen Fibres Orientation in the Bone Matrix around Dental Implants: Does the Implant’s Thread Design Play a Role?

**DOI:** 10.3390/ijms22157860

**Published:** 2021-07-23

**Authors:** Francesco Valente, Antonio Scarano, Giovanna Murmura, Giuseppe Varvara, Bruna Sinjari, Federico Mandelli, Maurizio Piattelli, Sergio Caputi, Tonino Traini

**Affiliations:** 1 Department of Innovative Technologies in Medicine & Dentistry, University “G. d’Annunzio” of Chieti-Pescara, 66100 Chieti, Italy; francesco.valente@unich.it (F.V.); antonio.scarano@unich.it (A.S.); giovanna.murmura@unich.it (G.M.); gvarvara@unich.it (G.V.); b.sinjari@unich.it (B.S.); maurizio.piattelli@unich.it (M.P.); sergio.caputi@unich.it (S.C.); 2 Electron Microscopy Laboratory, University “G. d’Annunzio” of Chieti-Pescara, 66100 Chieti, Italy; 3 Oral Surgery Specialist, Private Practice, 20145 Milan, Italy; federico.mandelli@gmail.com

**Keywords:** dental implant, thread shape, collagen fibres orientation, immediate loading, birefringence analysis, circularly polarized light

## Abstract

The aim of this study was to analyse the influence of different thread shapes of titanium dental implant on the bone collagen fibre orientation (BCFO) around loaded implants. Twenty titanium dental implants, divided for thread shapes in six groups (A–F) were analysed in the present study. All implants were immediately loaded and left in function for 6 months before retrieval. The parameters evaluated under scanning electron microscope were the thread width, thread depth, top radius of curvature, flank angle, and the inter-thread straight section. Two undecalcified histological sections were prepared from each implant. Birefringence analysis using circularly polarized light microscopy was used to quantitively measure BCFO. For groups A–F, respectively, transverse BCFO was 32.7%, 24.1%, 22.3%, 18.2%, 32.4%, and 21.2%, longitudinal BCFO was 28.2%, 14.5%, 44.9%, 33.1%, 37.7%, and 40.2%. The percentage differences between transverse and longitudinal orientation were 4.50% (A), 9.60% (B), −22.60% (C), −14.90% (D), −5.30% (E), and −19.00% (F). Following loading, the amount of transverse and longitudinal BCFO were significantly influenced by the thread shape. The greater flank angles and narrower inter-thread sections of the “V” shaped and “concave” shaped implant threads of groups A and B, respectively, promoted the predominance of transverse BCFO, compared to groups C-F (*p* < 0.05). A narrow inter-thread straight section promotes transverse BCFO, as do “V” shaped and “concave” shaped threads, which can thus be considered desirable design for implant threads.

## 1. Introduction

There are many dental implant types with several thread designs available on the market. The evolution of their design has seen incremental changes in size, shape, materials, and surfaces with respect to earlier designs. However, this has been prompted at times by market demands, rather than by any basic science research [1]. Therefore, considering the expanded indications for implants and changing clinical protocols, the relation between implant design and load distribution at the implant–bone interface has become an important issue.

According to the ‘mechanostat’ theory [2], bone has a mechanical competence that is related to the typical load peak. Therefore, the bone structure depends on the daily loading/stress patterns and the subsequent adaptation mechanism. This means that to achieve mechanical bone competence, the bone undergoes either ‘modelling’ and/or ‘remodelling’ processes. Continuous micro damage caused by repeated bone strain can activate bone reparative processes, which also influences the bone structure [3]. Similar effects on bone mass can be obtained by varying the magnitude and frequency of such a stimulus [4]. Understanding how threshold stress/strain values can affect bone responses is therefore important because, as Frost noted, “these thresholds are like the thermostats that switch a room’s heating and cooling mechanisms on and off, where ‘heating’ is analogous to increasing a bone’s strength by modelling, and ‘cooling’ is like decreasing a hollow bone’s strength by disuse-mode remodelling” [5].

Dental implants are devices that transmit loading forces from the dental arches to the jaw bones. Bone stability around the margins of fixtures is one of the key factors for long-term implant success, and this follows the same rules as for other load-bearing bones. Based on finite element analysis, the highest loading stress is concentrated in the region adjacent to the first thread of the implant [6]. Thus, Wang et al. concluded that the shape of the implant thread profile can have profound effects upon the magnitude of the stress that is transferred to the bone, and that very small threads of a favourable profile might be more effective [4]. On the other hand, it has been suggested that low-intensity mechanical signals and strains are strong determinants of bone morphology [7]. As bone does not always have the same density, another finite-elements study showed that the Young’s modulus of bone can affect the transmission of strain through trabecular bone [8].

Following classical histology, in terms of the collagen fibre organization, bone tissue can be classified into woven and lamellar bone. Woven bone is formed rapidly in response to wounding or hypertrophic adaptation, while remodelling processes give rise to lamellar bone formation. The main factors that determine the mechanical properties of bone appears to be related to both the presence of a collagen fibres orientation and the degree of mineralization of the matrix. The presence of oriented collagen fibres is particularly important in terms of the amount of energy required to cause matrix failure, while the stiffness of bone is related to the mineral content. The bone collagen fibre orientation (BCFO) is strongly linked to the loading regimen, as the load has a profound effect on the spatial orientation of the collagen fibres. Transversely oriented collagen fibres show the best resistance to compression strength, while longitudinally oriented collagen fibres show the best resistance to shear and traction strengths [4].

There are several advantages associated with externally threaded dental implants. The threads improve the primary stability of the implant, which reduces the micro-motion after implant insertion and during the healing period. In addition, forces applied to the implant body during function are spread across normal and tangential directions to the surface of the thread, and they are concentrated in certain locations [9].

In the past years, our research group evaluated the topic of BCFO in relation to implant threads and implant loading [10,11,12,13,14,15,16,17]. In humans, significant increases in transverse BCFO can be seen around loaded fixtures, compared to the surrounding alveolar bone [10]. These studies also reported significant increases in transverse BCFO around immediately loaded dental implants, as compared to unloaded implants, where significant increases in longitudinal BCFO were seen [11]. The predominance of transverse BCFO was also noted around an overloaded fractured dental implant after 5 years of function [12]. Dental implants placed under immediate loading conditions demonstrated significant increases in transverse BCFO after both 4 months and 12 years of loading [13]. In contrast, around unloaded dental implants, there was a predominance of longitudinal BCFO [14], along with low mineral density [15]. These results were also confirmed in animal models, where significant increases in transverse BCFO were seen around immediately loaded dental implants, as compared to unloaded ones [16]. Moreover, a relationship between functional loading and increases in transverse BCFO in terms of inter-implant distance was defined [17]. These studies thus demonstrated that biting forces applied to implants can have profound effects on the spatial organization of the collagen fibres within the bone matrix, with high concentrations of transverse BCFO associated with the lower flanks of implant threads.

The purpose of the present study was to determine the influence of different shapes of implant threads on the orientation of the collagen fibres within the bone matrix. In particularly, we aimed to evaluate the amount of transverse BCFO as a function of the shape of the implant thread. The null hypothesis states that there are no statistically significant differences in BCFO related to thread shape.

## 2. Results

**Figure 1:**
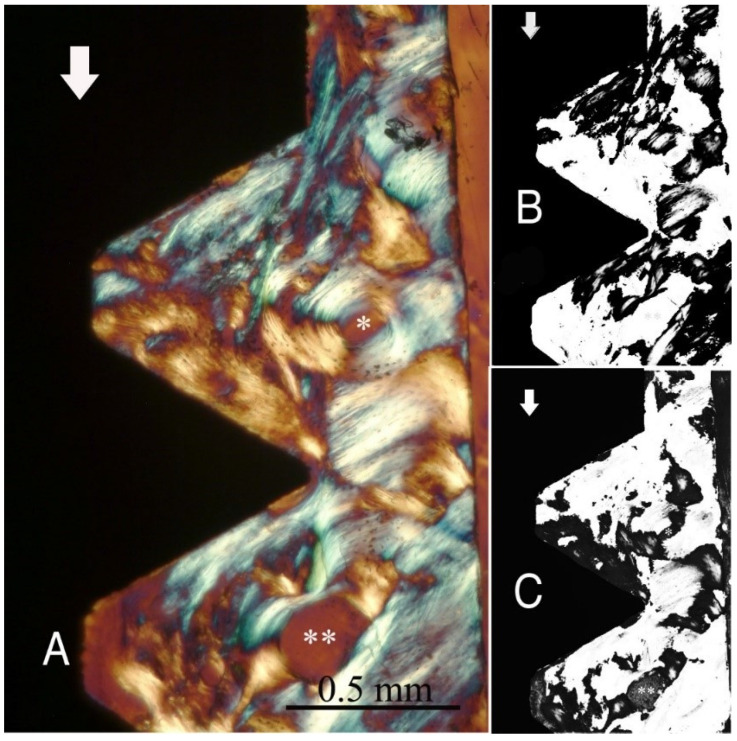
(**A**) Circularly polarized light microscopy image of unstained section for bone collagen fibre orientation evaluation around the implant threads of group A (see Table 1 for groups’ identification according to implant thread profile parameters). Mag. 100×. White arrows: main direction of occlusal load; *, **: Havers canals. The collagen fibre orientation of the bone adjacent to the lower flank and near the tip of the ‘V’-shaped threads is predominantly transverse (white-blue), while in other areas, it is longitudinal (yellow-orange). (**B**,**C**) Computer separations of transverse (**B**) and longitudinal (**C**) bone collagen fibre orientations.

**Figure 2:**
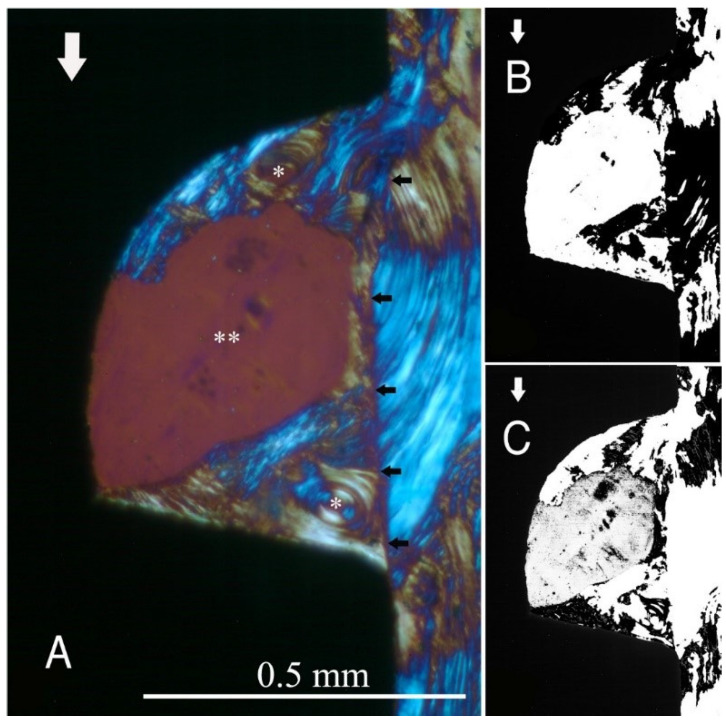
As for Figure 1, for group B (see Table 1). Mag. 100×. (**A**) White arrows: main direction of occlusal load; *: Havers canals; **: marrow space; black arrows: border between newly formed bone (left) and native bone (right). With the lower flank angle and near the tip of the thread, the bone collagen fibre orientation is transverse (white-blue), while in other areas, it is longitudinal (yellow-orange). (**B**,**C**) Computer separation of transverse (**B**) and longitudinal (**C**) bone collagen fibre orientations.

**Figure 3:**
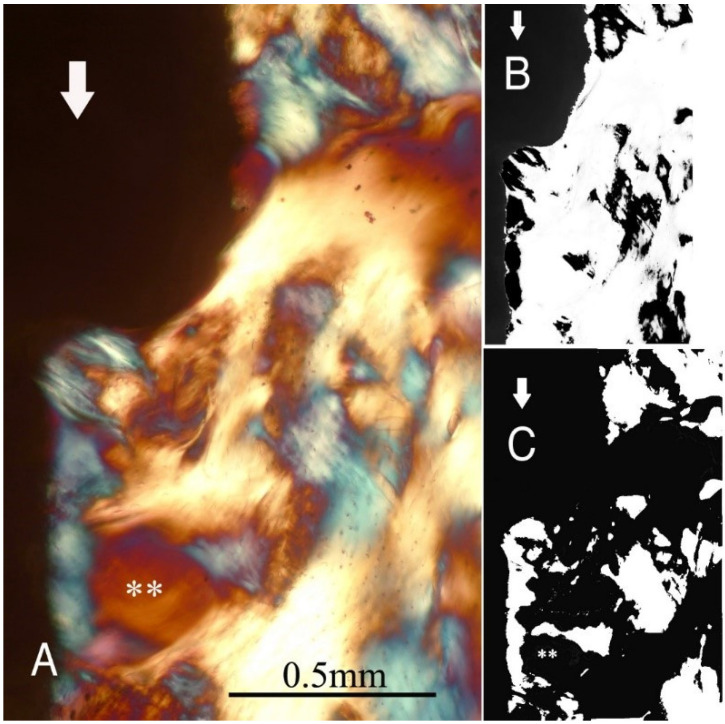
As for Figure 1, for group C (see Table 1). Mag. 100×. (**A**) White arrows, main direction of occlusal load; **: marrow space. The bone collagen fibre orientation is predominantly transverse only under the lower flank of the small thread and partially along the straight section (white-blue), while in other areas, it is longitudinal (yellow-orange). (**B**,**C**) Computer separation of transverse (**B**) and longitudinal (**C**) bone collagen fibre orientations.

**Figure 4:**
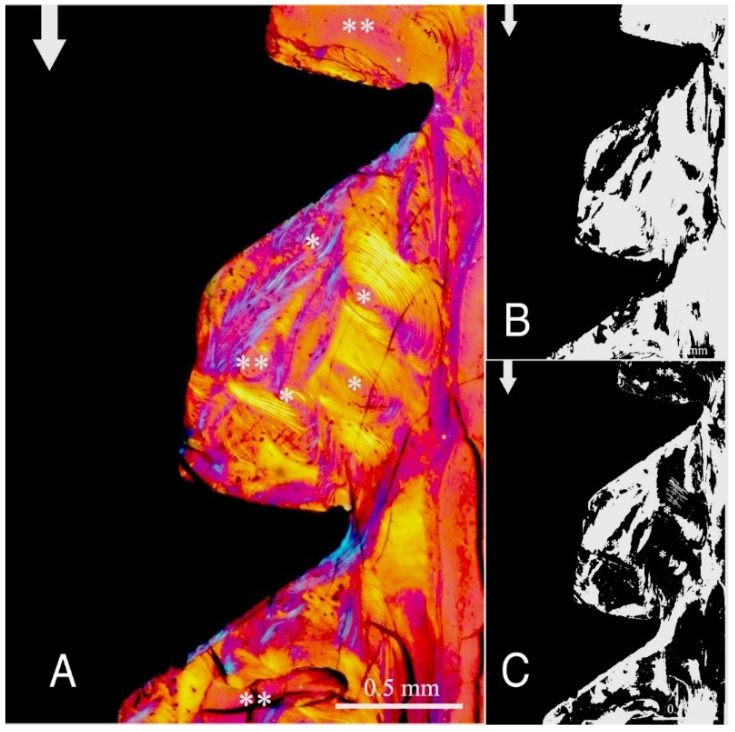
As for Figure 1, for group D (see Table 1). Mag. 100×. (**A**) White arrows: main direction of occlusal load; *: Havers canals; **: marrow space. The bone collagen fibre orientation adjacent to the lower flank of the threads is predominantly transverse (white-blue), while in other areas, it is longitudinal (yellow-orange). (**B**,**C**) Computer separation of transverse (**B**) and longitudinal (**C**) bone collagen fibre orientations.

**Figure 5:**
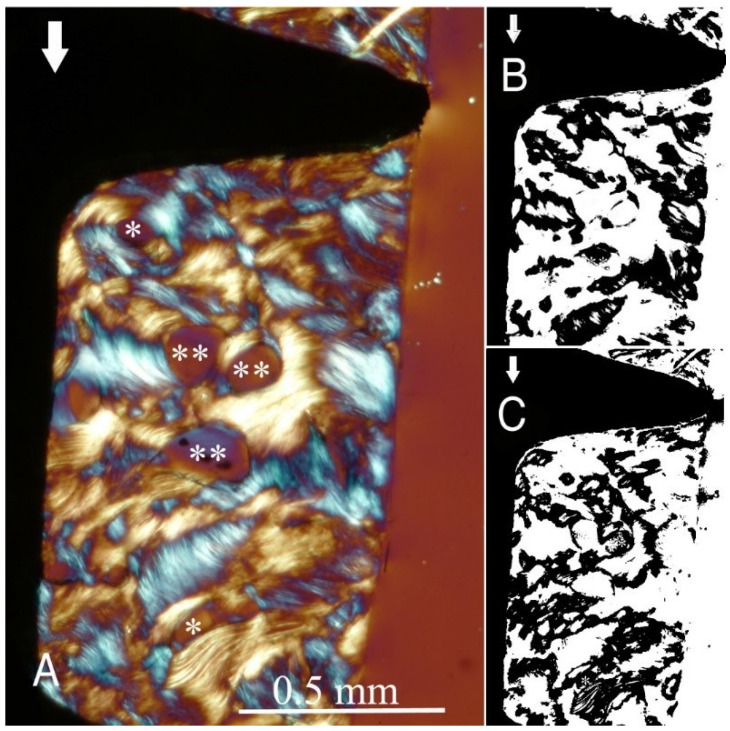
As for Figure 1, for group E (see Table 1). Mag. 100×. (**A**) White arrows: main direction of occlusal load; *: Havers canals; **: marrow space. The bone collagen fibre orientation is transverse (white-blue) and longitudinal (yellow-orange). (**B**,**C**) Computer separation of transverse (**B**) and longitudinal (**C**) bone collagen fibre orientations.

**Figure 6:**
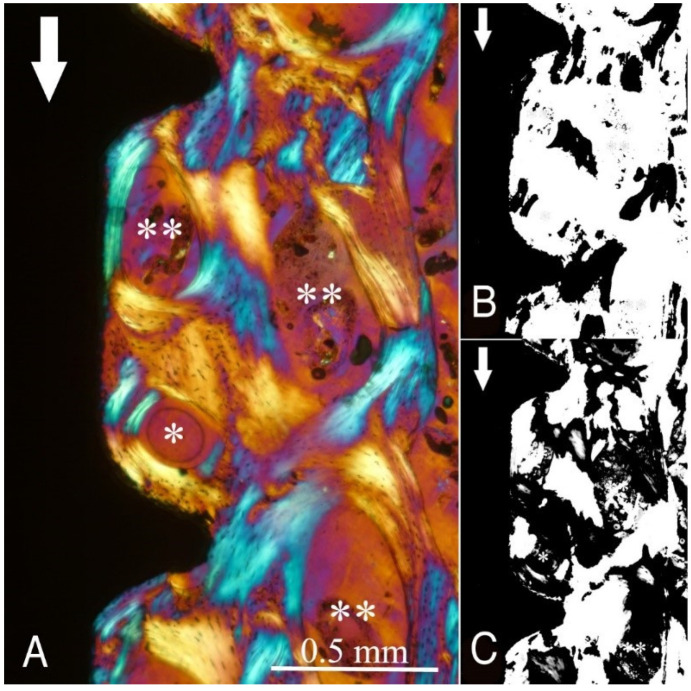
As for Figure 1, for group F (see Table 1). Mag. 100×. (**A**) White arrows: main direction of occlusal load; *: Havers canals; **: marrow space. The bone collagen fibre orientation is transverse (white-blue), and longitudinal (yellow-orange). (**B**,**C**) Computer separation of transverse (**B**) and longitudinal (**C**) bone collagen fibre orientations.

The study groups design used in this study is displayed in Table 1 and it is comprehensively described in Section 4 (Materials and Methods). The relative amount and the differences between the transverse and longitudinal collagen fibres in the bone samples of each group are summarized in Table 2 and illustrated in Figure 1, Figure 2, Figure 3, Figure 4, Figure 5 and Figure 6 and Figure S1. Of further note, for group A (Figure 1) and group B (Figure 2), the transversely oriented collagen fibres were mainly seen adjacent to the lower flank of the thread of the dental implants, while the longitudinally oriented collagen fibres were more spread throughout the bone tissue. Figure 3, Figure 4, Figure 5 and Figure 6 illustrate the bone samples for groups C to F, respectively.

**Figure 7:**
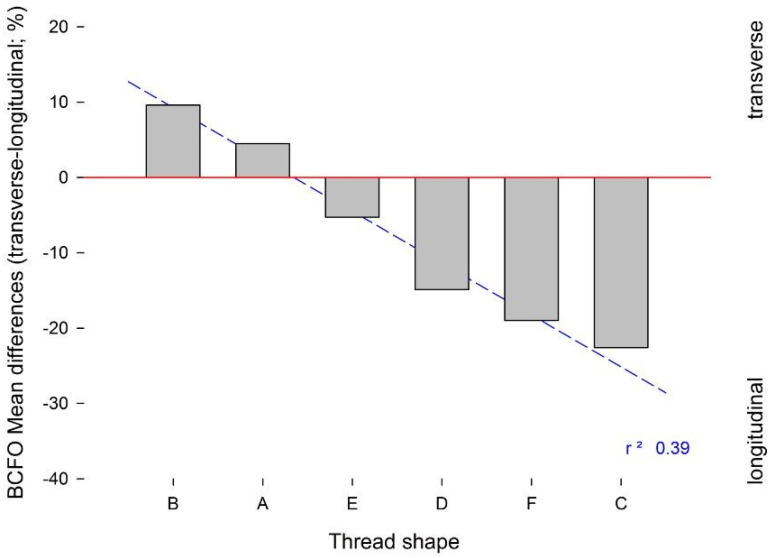
Linear regression analysis of means differences for bone collagen fibre orientation (BCFO), transverse minus longitudinal. The regression line shows a significant decrease in transverse BCFO and increase in longitudinal BCFO in the groups with narrow threads and wider inter-thread straight section (groups C-F).

The results of the statistical analysis are summarized in Table 3 and Table 4. Here, the “V”-shaped and “concave”-shaped implant threads of groups A and B showed predominance of transverse BCFO, compared to groups C, D, E, and F (*p* < 0.05). Similarly, the linear regression analysis showed predominance of longitudinal BCFO for groups C, D, E, and F (Figure 7). Here, the presence of a wide inter-thread straight section was less favourable to the development of transverse BCFO near the implant surface.

## 3. Discussion

The results of the present investigation reject the hypothesis under test, as the shape of the implant threads had a significant influence on the bone microstructure. Across the different implant designs, an interesting relationship between the depth of the implant thread and the amount of transverse BCFO can be appreciated, while a wide inter-thread section of an implant appears to be related to increased longitudinal BCFO. This last aspect should be considered for cylindrical dental implants and for the implant design, as for group E, where even if the presence of a wide inter-thread section (1.2 mm) would have a positive effect on longitudinal BCFO, the association with a deep thread (0.9 mm) minimized the difference between transverse and longitudinal BCFO (−5.30 ± 3.50%). Another interesting outcome is appreciable in group D, where a similar amount of thread depth (0.7 mm) to group E and an inter-thread section of 0.8 mm instead produced inferior BCFO mean difference (−14.90 ± 3.50%), although the differences between the groups were not statistically significant. In this group, aside from the other parameters, the increased flank angle (37°) may have influenced the transversal–longitudinal BCFO difference. The groups showing the highest mean difference between transverse and longitudinal BCFO were group A (4.50 ± 3.95%) and group B (9.60 ± 5.00%), with statistically significant differences between nearly all the other groups. This highlight how the “V”-shaped thread of group A and the “concave” shaped thread of group B are the most favourable design of the externally threated implant to stimulate the BCFO able to bear the occlusal load more effectively. The “V” shaped thread design is characterised by a triangle-shaped thread design with a pointed end; the characteristic of the “concave”-shaped thread is the concave profile of the lower flank. In particular, the outcome of the “concave” shape reflects the findings by Ripamonti et al., who found on a primate model that the surface geometry of hydroxyapatite (HA)-coated implants was important in the bone formation and that bone formation can initiate in concavities rather than on convexities of the implant surface. It seems that concavities provide a suitable environment for bone formation, possibly due to mechanical forces, blood clot retention, and the presence or gradients of chemotactic and other agents from the healing process [18]. It is interesting to note that the common parameter of these two kinds of thread design is the narrower inter-thread section, with respect to the other groups, and it can also possibly play a role in the promotion of the transverse BCFO.

Most of the histological evidence in implant dentistry has been based on the relative bone–implant contact rate, without any consideration of the bone matrix organization, and particularly for BCFO. However, it now needs to be considered that the long-term survival of osseointegrated implants is more dependent on the effective functioning of the bone under occlusal loads rather than on the level of contact between the peri-implant bone tissue and the implant surface. Thus, all the microstructural features that influence the mechanical properties of the bone, such as the spatial arrangement of the collagen fibres and the bone mineral content, should be considered as very important factors [15].

The loading environment of the bone affects the collagen orientation: bone under compression shows oblique/transversely oriented collagen fibres, while that under traction shows longitudinally oriented collagen fibres [10,11,12,13,14,15,16]. It has been reported that the local mechanical environment around implants is dependent on the forces imposed and the implant/bone surface interaction, along with certain implant designs [19]. Moreover, the use of an external thread can result in significant variations along the bone–implant interface [20,21].

To the best of our knowledge, this is the first study that has considered the effects of different implant thread shapes in the analysis of bone organization and microstructure in humans. Instead, in the dental literature, the few studies on this topic were performed using finite element analysis, which raises some concerns due to the models of investigation [22]. Indeed, finite element analysis is not an accurate method to describe stress/strain development at the bone–implant interface, as it is not possible to consider other factors at the same time, such as the bone anisotropy, microstructural organization (i.e., BCFO, bone mineral density), and physiology (i.e., modelling and remodelling). As a consequence, previous data have often appeared inconsistent with clinical and biological evidence [23].

Although the present study and the data generated here should be considered preliminary at this stage, they contribute towards the development of an ideal model for dental implants. However, the evaluation here is incomplete, as other biological aspects such as basic bone physiology and bone modelling and remodelling should also be taken into account to determine the final “favourable” versus “unfavourable” implant thread shape. Another main aspect towards which future studies should be directed is the in vivo assessment in humans of each parameter determining the thread architecture (thread depth, width, inter-thread section, flank angle, top radius of curvature, etc.) in influencing the BCFO, beyond the given macro-structure of the implant external thread. The main limitation of the present study relates to the small sample size of the groups; nevertheless, at the same time, the extreme importance of such samples retrieved from humans must be considered.

## 4. Materials and Methods

**Figure 8:**
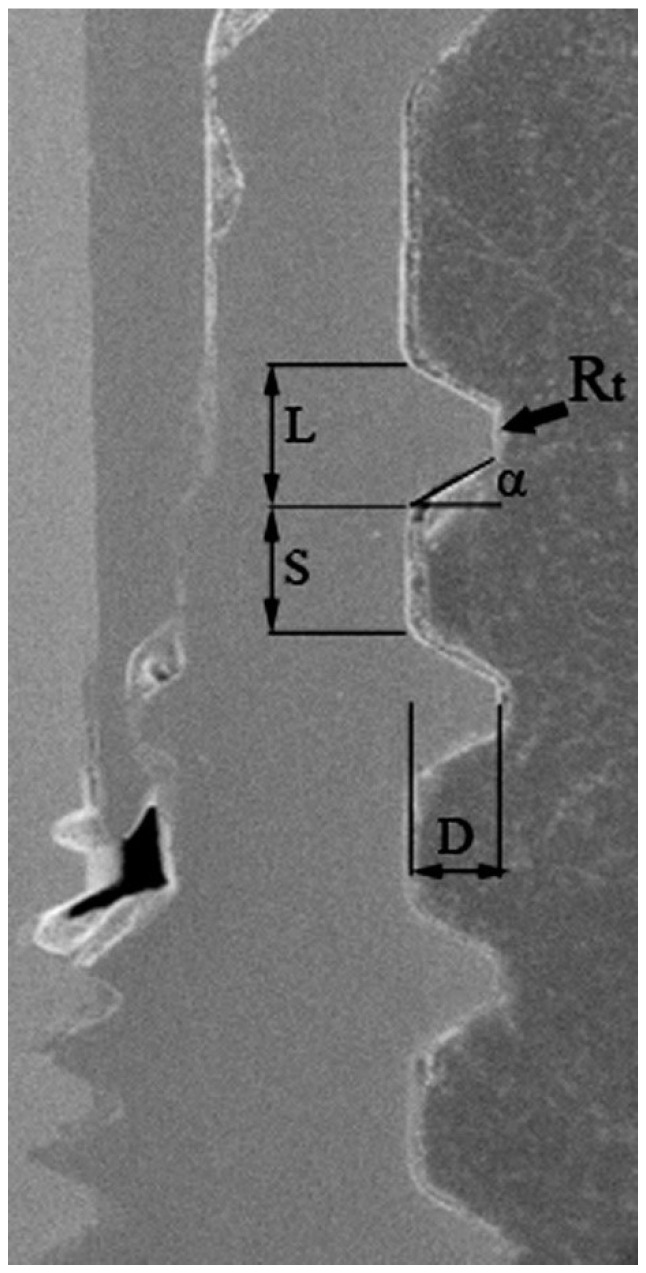
SEM image for definition of the titanium dental implant thread design, according to the thread width (L), thread depth (D), top radius of thread curvature (Rt), flank angle (α), and inter-thread straight section (S). Implant on the left, bone on the right. Mag. 50×.

A total of twenty titanium dental implant specimens were considered in the present study. Six different types of dental implants were assigned to groups named A–F (Figure 8), based on the characteristics of the thread profile shape. The different thread design parameters measured through scanning electron microscopy (SEM) (Zeiss EVO 50 XVP; Carl Zeiss AG, Oberkochen, Germany) are summarized in Table 1. All the specimens were part of different human studies that have already been published, where the specific surgical protocols and details about the manufacturer, length, and diameter of the implants can be found [10,11,12,13,14,15]. All the implants were immediately loaded and left in function for 6 months, before their retrieval.

The present study was conducted in full compliance with ethical principles, including those of the World Medical Association Declaration of Helsinki. The patients involved in each study protocol gave their written informed consent [10,11,12,13,14,15]. Protocols were approved by the Ethics Committee of University of Chieti (Project identification codes: 2604, January 2006; 01, April 2007; 2701, year 2008).

### 4.1. Histological Examination

After retrieval, the bone specimens were fixed in 10% buffered formalin, rinsed, and dehydrated through an ascending series of alcohol before being embedded in glycol methacrylate resin (Techonovit 7200 VLC; Kulzer, Wehrheim, Germany). After polymerization, the specimens were sectioned (100 ± 20 µm) along their longitudinal axis using a high-precision diamond disc, and then ground down to 50 ± 5 µm with custom-built sawing and grinding apparatus (TT System; TMA2, Grottammare, Italy). The specimens were left unstained to be investigated under polarized light.

### 4.2. Circularly Polarized Light Microscopy

Birefringence under circularly polarized light microscopy was used to evaluate BCFO around the implants. In brief, birefringence (*B_f_*) makes use of the refractive index, *n*, of a material, as defined in Equation (1):

(1) $$B_{f}=H+\frac{I\lambda^{2}}{\lambda^{2}-G}+\frac{J\lambda^{2}}{\lambda^{2}-L}$$

where *G*, *H*, *I*, *J* and *L* are the dispersion coefficients for birefringence of the optical materials and *λ* is the wavelength in microns. Birefringence spectroscopy is the optical technique of measuring orientation in an optically anisotropic sample by measuring the retardation of polarized light passing through the sample.

Retardation Γ is given by Equation (2):

(2) $$\Gamma =t{\left(n_{e}-n_{0}\right)}_{i}$$

where *t* is the thickness of bone in the section; *n_e_* and *n*_0_ are the refractive indices of the extraordinary and ordinary rays; the term (*n_e_ − n*_0_)*_i_* is called the intrinsic birefringence and is a characteristic property of the tissue dependent on the molecular alignment and the orientation and nature of the chemical. If the molecules and bonds had a random organization, the refractive indices of the two rays would be equal and the retardation would be zero. However, as the degree of alignment of polypeptide chains and chemical bonds in the collagen increases, the difference in the refractive index of the two rays increases; hence, the retardation increases. Two levels of optical retardations (OR) values were estimated when the collagen fibres were positioned at ±45° relative to the “plane of polarized light”. OR was measured by the Senarmont method, using a λ/4 compensator and monochromatic light (*λ* = 546 nm) obtained by a narrow band-pass interference filter (Edmund Industrial Optics, Barrington, IL, USA).

Although circularly polarized light microscopy can discriminate BCFO differences of 5°, under the present evaluation conditions, only the transverse and longitudinal orientations were considered here, as these two orientations are strictly related to the bone strain direction, as reported by Riggs et al. [24] and Wang et al. [4]. A transmitted brightfield light microscope (Axiolab, Zeiss Oberchen, Jena, Germany) connected to a high-resolution digital camera (FinePix S2 Pro, Fuji Photo Film Co. Ltd., Minato-Ku, Japan) was used, and it was equipped with two linear polarizers and two quarter-wave plates arranged to transmit circularly polarized light. For each specimen, two unstained sections of 50 μm thickness were used. The area of analysis for all specimens comprised the bone adjacent to the first two osseointegrated implant threads. All images were collected at 100× magnification and examined using Image-Pro Plus version 6.0 (Media Cybernetics Inc., Bethesda, MD, USA). To ensure accuracy, the software was calibrated for each experimental image using a software feature named ‘Calibration Wizard’, which reports the number of pixels between two selected points (diameter or length of the implant). The linear remapping of the pixel numbers was used to calibrate the distance in micrometres. The transversally and longitudinally oriented collagen-bundle areas were expressed as a percentage of the total bone area considered.

### 4.3. Statistical Analysis

The statistical analysis was carried out using Sigma Stat 3.5 statistical package (SPSS Inc., Ekrath, Germany). The differences were evaluated using parametric tests, as the data were normally distributed. Statistical evaluation was carried out using one-way ANOVA to test for overall significance, followed by Holm–Sidak tests for multiple comparisons among the groups to identify individually significant data. Linear regression analysis was conducted to investigate the correlation between the amount of transverse and longitudinal BCFO and thread shape. A *p* value < 0.05 was considered the threshold to detect statistically significant differences.

## 5. Conclusions

Within the limitations of this study, it can be concluded that around the dental implants, the amount of transverse and longitudinal BCFO were significantly influenced by the thread shape. Both the “V”-shaped and “concave”-shaped implant threads showed the best BCFO arrangements, as they provided a prevalence of transverse collagen fibres, which can be considered a positive prognostic factor for long-term clinical implant success.

## Figures and Tables

**Table 1:** Groups’ definition through parameters of titanium dental implant thread profiles considered in this study.

Group	Thread Characteristic (mm)			Flank Angle (°)	Inter-Thread Section (mm)
	Width	Depth	Top Radius of Curvature		
A	0.8	0.6	0.4	30	0.2
B	0.6	0.4	0.6	35	0.4
C	0.9	0.5	0.7	7	1.4
D	0.2	0.7	0.2	37	0.8
E	0.5	0.9	0.8	10	1.2
F	0.4	0.3	0.6	31	1.2

**Table 2:** Relative levels of transverse and longitudinal collagen fibres in the bone tissue, as revealed by birefringence under circularly polarized light microscopy.

Group	Transverse Collagen Fibres (%)	Longitudinal Collagen Fibres (%)	Difference (Trans.-Long.) (%)
A	32.7 ± 3.6	28.2 ± 4.3	4.50 ± 3.95
B	24.1 ± 4.7	14.5 ± 5.3	9.60 ± 5.00
C	22.3 ± 3.2	44.9 ± 4.8	−22.60 ± 4.00
D	18.2 ± 2.9	33.1 ± 4.1	−14.90 ± 3.50
E	32.4 ± 3.1	37.7 ± 3.9	−5.30 ± 3.50
F	21.2 ± 4.3	40.2 ± 5.1	−19.00 ± 4.70

**Table 3:** One-way ANOVA.

Group	N	Mean	SD	SEM	
A	3	4.50	3.95	2.28	
B	6	9.60	5.00	2.04	
C	4	−22.60	4.00	2.00	
D	3	−14.90	3.50	2.02	
E	2	−5.30	3.50	2.47	
F	2	−19.00	4.70	3.32	
**Source of Variation**	**DF**	**SS**	**MF**	**S**	***p***
Between groups	5	3467.022	693.404	39.905	<0.001
Residual	14	263.045	18.789		
Total	19	3730.067			

SD: standard deviation, SEM: standard error of the means, DF: degree of freedom, SS: sum of square, MS: mean of square, F: index, *p*: significance. The differences in the means among the treatment groups were greater than would be expected by chance (*p* < 0.001). Power of performed test with alpha = 0.050:1.000.

**Table 4:** Pairwise multiple comparisons (Holm–Sidak method).

Comparison	Difference of Means	Critical Level	Significance (*p* < 0.05)
B vs. C	32.200	0.003	Yes
A vs. C	27.100	0.004	Yes
B vs. F	28.600	0.004	Yes
B vs. D	24.500	0.004	Yes
A vs. F	23.500	0.005	Yes
E vs. C	17.300	0.006	Yes
B vs. E	14.900	0.006	Yes
E vs. F	13.700	0.007	Yes
A vs. E	9.800	0.009	No
E vs. D	9.600	0.010	No
E vs. C	7.700	0.013	No
B vs. A	5.100	0.017	No
D vs. F	4.100	0.025	No
F vs. C	3.600	0.050	No

## Data Availability

Data are available upon request to the corresponding author.

## Supplementary Materials

The following are available online at https://www.mdpi.com/article/10.3390/ijms22157860/s1.
