# Supplementary material for: Collagen Fibres Orientation in the Bone Matrix around Dental Implants: Does the Implant’s Thread Design Play a Role?
Source: Int J Mol Sci. 2021 Jul 23;22(15):7860. doi: 10.3390/ijms22157860 (PMC8346163; doi:10.3390/ijms22157860)
Supplement: Supplementary file 1 [file ijms-22-07860-s001.zip › ijms-1279485-supplementary.pdf]

International Journal of
Molecular Sciences

MDPI

Article

Collagen Fibres Orientation in the Bone Matrix Around Dental Implants: Does the Implant's Thread Design Play a Role?

Francesco Valente [1,2], Antonio Scarano [1], Giovanna Murmura [1], Giuseppe Varvara [1], Bruna Sinjari [1,2], Federico Mandelli [3], Maurizio Piattelli [1], Sergio Caputi [1,2] and Tonino Traini [1,2,*]

[1] Department of Innovative Technologies in Medicine & Dentistry, University "G. d'Annunzio" of Chieti-Pescara, 66100 Chieti, Italy; francesco.valente@unich.it (F.V.); antonio.scarano@unich.it (A.S.); giovanna.murmura@unich.it (G.M.); gvarvara@unich.it (G.V.); b.sinjari@unich.it (B.S.); maurizio.piattelli@unich.it (M.P.); sergio.caputi@unich.it (S.C.)
[2] Electron Microscopy Laboratory, University "G. d'Annunzio" of Chieti-Pescara, 66100 Chieti, Italy
[3] Oral Surgery Specialist, Private Practice, 20145 Milan, Italy; federico.mandelli@gmail.com
* Correspondence: tonino.traini@unich.it; Tel.: +39-08713554143

Supplementary Material

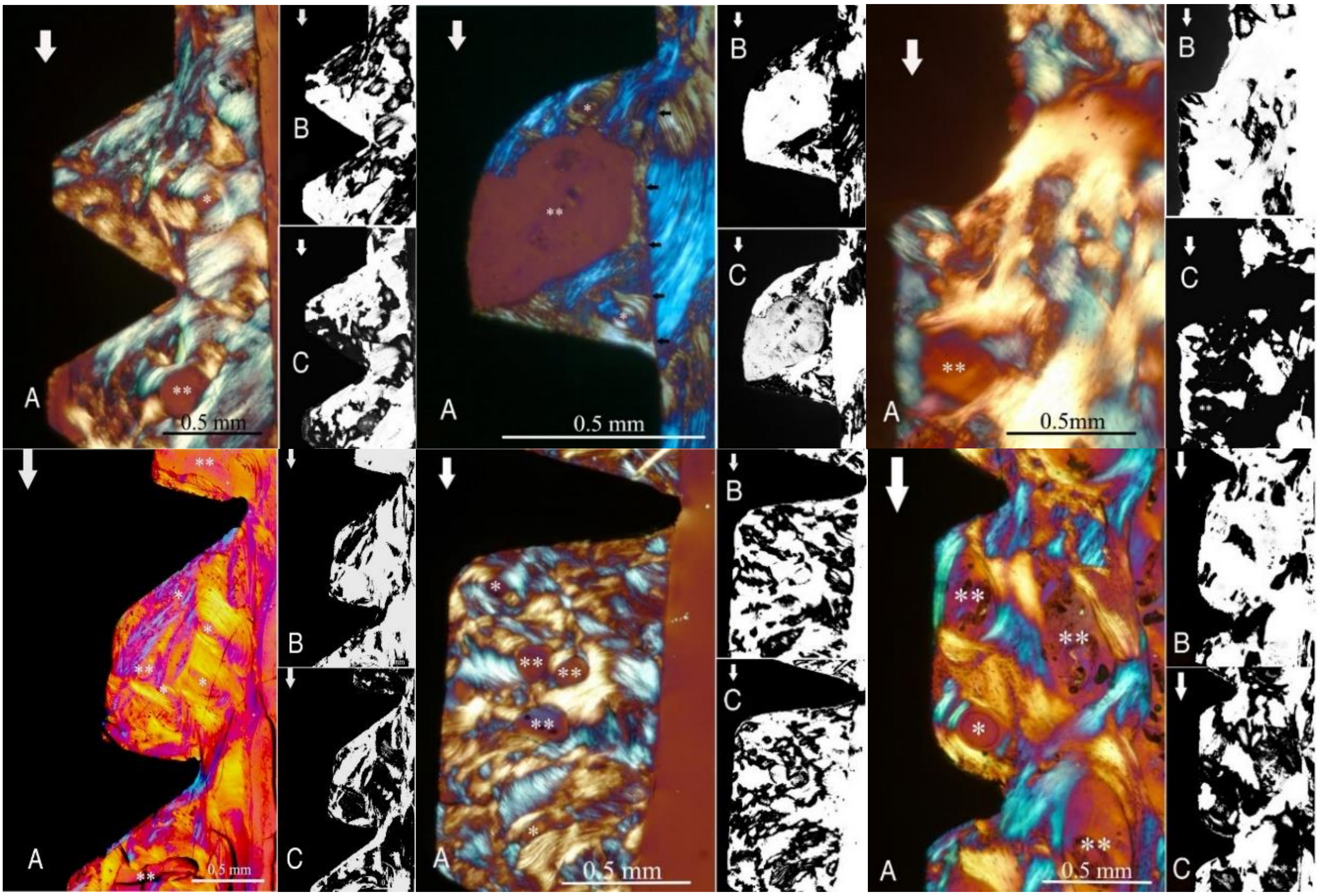

Figure S1: Figure 1 to 6 collected for a simplified comparison.
